# Current Status and Future Perspectives of Perioperative Therapy for Resectable Biliary Tract Cancer: A Multidisciplinary Review

**DOI:** 10.3390/cancers13071647

**Published:** 2021-04-01

**Authors:** Changhoon Yoo, Sang Hyun Shin, Joon-Oh Park, Kyu-Pyo Kim, Jae Ho Jeong, Baek-Yeol Ryoo, Woohyung Lee, Ki-Byung Song, Dae-Wook Hwang, Jin-hong Park, Jae Hoon Lee

**Affiliations:** 1 Department of Oncology, Asan Medical Center, University of Ulsan College of Medicine, Seoul 05505, Korea; kkp1122@amc.seoul.kr (K.-P.K.); jaeho.jeong@amc.seoul.kr (J.H.J.); ryooby@amc.seoul.kr (B.-Y.R.); 2 Department of Surgery, Samsung Medical Center, School of Medicine, Sungkyunkwan University, Seoul 06351, Korea; surgeonssh@gmail.com; 3 Division of Hematology and Oncology, Department of Internal Medicine, Samsung Medical Center, School of Medicine, Sungkyunkwan University, Seoul 06351, Korea; oncopark@skku.edu; 4 Department of Surgery, Asan Medical Center, College of Medicine, University of Ulsan, Seoul 05505, Korea; ywhnet@gmail.com (W.L.); mtsong21c@naver.com (K.-B.S.); dwhwang@amc.seoul.kr (D.-W.H.); 5 Department of Radiation Oncology, Asan Medical Center, College of Medicine, University of Ulsan, Seoul 05505, Korea; pjhynwie@hanmail.net

**Keywords:** biliary tract cancer, cholangiocarcinoma, gallbladder cancer, adjuvant chemotherapy, radiation therapy, surgery

## Abstract

**Simple Summary:**

For decades, there has been no globally accepted neoadjuvant or adjuvant therapy in resectable biliary tract cancer. Based on the results of the BILCAP trial, adjuvant capecitabine has been widely regarded as standard adjuvant therapy. Focusing on the management of resectable biliary tract cancer, this article reviews each therapeutic strategy including surgery, chemotherapy and radiotherapy, and summarises published and ongoing clinical trials of neoadjuvant and adjuvant therapy.

**Abstract:**

Biliary tract cancers (BTCs) are a group of aggressive malignancies that arise from the bile duct and gallbladder. BTCs include intrahepatic cholangiocarcinoma (IH-CCA), extrahepatic cholangiocarcinoma (EH-CCA), and gallbladder cancer (GBCA). BTCs are highly heterogeneous cancers in terms of anatomical, clinical, and pathological characteristics. Until recently, the treatment of resectable BTC, including surgery, adjuvant chemotherapy, and radiation therapy, has largely been based on institutional practice guidelines and evidence from small retrospective studies. Recently, several large randomized prospective trials have been published, and there are ongoing randomized trials for resectable BTC. In this article, we review prior and recently updated evidence regarding surgery, adjuvant and neoadjuvant chemotherapy, and adjuvant radiation therapy for patients with resectable BTC.

## 1. Introduction

**Figure 1:**
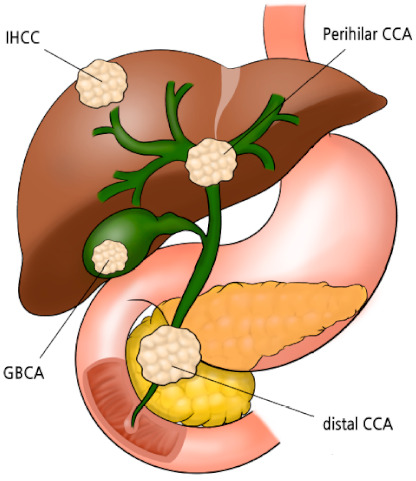
Structural classification of biliary tract cancer, IHCC = intrahepatic cholangiocarcinoma, CCA = cholangiocarcinoma, and GBCA = gallbladder cancer.

Biliary tract cancers (BTCs) are a group of aggressive malignancies that arise from the bile duct and gallbladder. BTCs include intrahepatic cholangiocarcinoma (IH-CCA), extrahepatic cholangiocarcinoma (EH-CCA), and gallbladder cancer (GBCA) (Figure 1) [1]. EH-CCA is subdivided into perihilar and distal CCA. Globally, there are an estimated 186,000 new cases and 140,000 deaths annually due to BTCs. BTC has a particularly high incidence in some countries, including South Korea, Thailand, China, and Chile [1], but it is rare in most Western countries. However, its incidence is now increasing globally [1,2]. BTCs are highly heterogeneous cancers in terms of anatomical, clinical, and pathological characteristics. Patients with BTC often present with non-specific and non-biliary obstructive symptoms, which complicates and delays diagnosis, and only 20% of patients are diagnosed at the resectable stage [2,3]. BTCs are well known to have a dismal prognosis, with estimated 5-year overall survival (OS) rates of 6–26% for localized disease and 1–2% for metastatic disease [2,3]. For localized BTCs, surgical resection is the only curative treatment modality. The rate of BTC recurrence is high even after curative-intent surgical resection. For unresectable or metastatic BTCs, gemcitabine plus cisplatin (GemCis) is the standard or care based on the improved survival outcomes compared to gemcitabine monotherapy in the ABC-02 trial [4]. However, OS of patients with unresectable or metastatic BTC treated with GemCis is still dismal with less than median 1 year in the randomized trials and real-world data [4,5,6,7]. There is no globally established second-line chemotherapy after progression on GemCis, although fluorouracil-based therapy has been widely used in daily practice [8]. An improved recent understanding of genetic characteristics of BTC has increased the chances of incorporation of molecular targeted therapy in the management of unresectable or metastatic BTC such as fibroblast growth factor receptor (FGFR) inhibitors, isocitrate dehydrogenase (IDH)-1 inhibitor, and human epidermal growth factor receptor 2 (HER2) inhibitors [9,10,11,12]. However, only small subsets of patients up to 20% of overall BTC patients harbor these genetic alterations and there are discrepancies according to the primary tumor sites (FGFR2 gene fusion and IDH-1 in IH-CCA, and HER2 in GBCA or EH-CCA).

The high recurrence rates and dismal prognosis of localized BTCs indicate the unmet need for effective adjuvant and neoadjuvant therapy. However, most previous studies investigating adjuvant and neoadjuvant therapy have been based on small and retrospective analyses of heterogeneous patient populations. Recently, several large randomized prospective trials have been published, and there are ongoing randomized trials investigating resectable BTC [13,14,15]. In this article, we review prior and recently updated evidence regarding surgery, adjuvant and neoadjuvant chemotherapy, and adjuvant radiation therapy for patients with resectable BTC.

## 2. Surgical Considerations

Radical resection with microscopically negative margins is essential for curing BTC. Considering the structural characteristics of the biliary tree, from intrahepatic to the ampulla of Vater, selection of the optimal extent for surgical resection (according to various locations) is critically important. Additionally, appropriate lymph node dissection should be performed for proper treatment and staging.

### 2.1. IH-CCA

Based on the involved hepatic segment, the goals of surgical resection for IH-CCA are to perform margin-free hepatic resection and regional lymphadenectomy. Although it is still controversial whether to perform anatomical or non-anatomical resection for IH-CCA [16,17], achieving negative hepatic resection margins (R0 resection) has been reported as an important prognostic factor in terms of both recurrence and survival [18,19].

Although the necessity of routine regional lymphadenectomy remains controversial in terms of survival benefit, a lack of nodal staging may lead to heterogeneous and potentially incorrect prognostic classification [20,21,22]. The recently published eighth edition of the American Joint Committee on Cancer (AJCC) guidelines recommend routine regional lymphadenectomy, including the removal of at least six lymph nodes for appropriate staging [23]. Along with standard lymphadenectomy of the hepatoduodenal ligament (station 8) and hepatic artery (station 12), the recent evidence suggests additional lymphadenectomy of the left gastric artery nodes along the lesser curvature of the stomach in left-dominant IH-CCA and the retropancreatic area (station 13) in right-dominant IH-CCA [24,25].

### 2.2. GBCA

The principle of surgical resection for GBCA depends on the tumor depth of invasion (T stage of AJCC 8th edition). Simple cholecystectomy is sufficient for T1a tumors confined to the lamina propria, as the likelihood of finding residual disease in such tumors is almost negligible [26,27]. For T1b or T2 tumors, radical cholecystectomy with en-bloc resection of the adjacent liver parenchyma is recommended, along with hepatoduodenal lymphadenectomy. However, the optimal surgical strategy for T1b cancer invading the muscular layer is still under debate, and the role of radical resection for such tumors remains questionable [28,29,30]

Although there is still some controversy surrounding the treatment of stage T2a cancers, the current standard is to perform radical resection with limited or, in some advanced cases, extended hepatectomy and regional lymphadenectomy for GBCA beyond T2 invading perimuscular connective tissue [28,31]. Resection of the common bile duct with Roux-en-Y hepaticojejunostomy is not routinely recommended because it increases morbidity without any survival benefit. However, extrahepatic bile duct resection is necessary if the patient has a positive cystic duct margin on intraoperative frozen section or if there is direct tumor extension [32].

### 2.3. Perihilar CCA

**Figure 2:**
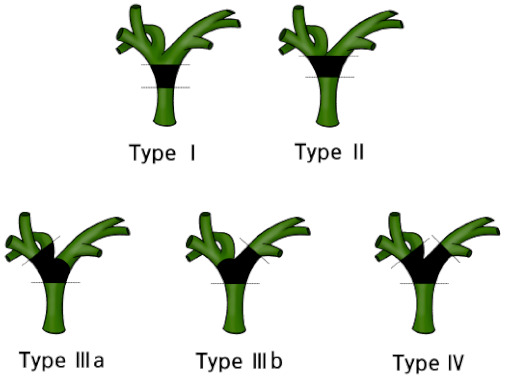
Bismuth–Corlette classification of perihilar cholangiocarcinoma, Type I, confined to the common hepatic duct; Type II, involved the confluence of the common hepatic duct; Type IIIa, involved the confluence and extended to the bifurcation of right hepatic duct; Type IIIb, involved the confluence and extended to the bifurcation of the left hepatic duct; and Type IV, involved the confluence and extended to the bifurcation of both right and left hepatic ducts.

Based on the mode of tumor extension, according to the Bismuth and Corlette classification, extended right or left hemihepatectomy, involving half of the liver, the inferior part of segment IV or V, most of the caudate lobe, the hilar plate, the extrahepatic bile duct, and regional lymphadenectomy, is regarded as the standard radical operation for perihilar CCA [33,34,35,36]. Even for patients with Bismuth and Corlette types I or II (Figure 2), extended hemihepatectomy is warranted to ensure negative margins and improve survival among patients suitable for surgery [37]. Type IV (Figure 2) is often classified as inoperable, but for some patients, right- or left-trisectionectomy is advantageous in terms of obtaining negative bile duct margins [38]. Combined vascular resection with reconstruction of the portal vein and hepatic artery could be applied to achieve R0 resection among patients with advanced tumors.

Perihilar CCA usually requires extended hepatectomy, which is related to the increased rate of postoperative hepatic failure and subsequent high rates of perioperative morbidity and mortality. Makuuchi et al. first introduced preoperative portal vein embolization for perihilar CCA with a small future remnant hepatic volume [39], and it is now widely accepted as a valuable preoperative measure that induces compensatory hypertrophy of the remnant liver parenchyma for extended hepatectomy [40,41,42].

### 2.4. Distal CCA

Pancreaticoduodenectomy (PD) with regional lymphadenectomy is the standard treatment for distal CCA. Pylorus preservation does not affect survival outcomes for distal CCA [43]. Standard regional lymphadenectomy, including hepatoduodenal ligament (station 8 and 12) lymph nodes and peripancreatic lymph nodes, is warranted, but extended lymphadenectomy, including para-aortic lymph nodes, is not justified in terms of survival benefit and perioperative morbidity [44,45].

## 3. Clinical Outcomes and Recurrence Patterns among Patients with Resected BTC

In a large single-center retrospective analysis including 564 resected BTC patients, the median OS was 28, 13, and 18 months among patients with IH-CCA, perihilar CCA, and distal CCA, respectively [19]. Among patients who underwent R0 resection, the median OS was 80, 30, and 25 months, respectively. Negative resection margin (i.e., R0 resection), negative lymph node status, and a higher degree of tumor differentiation were significantly better prognostic factors for resected BTC patients.

Another retrospective analysis, including 156 resected perihilar CCAs and GBCAs, showed that recurrence pattern varies by primary BTC site [46]. Locoregional recurrence is more frequent in association with perihilar CCA (65%) compared with GBCA (28%), while distant metastasis is dominant in GBCA (72%) compared with perihilar CCA (36%) as an initial recurrence site.

## 4. Adjuvant Chemotherapy

### 4.1. Meta-Analysis

Even as recently as 2010, there existed few prospective randomized trials investigating resectable BTC, and the mostly available evidence was data from small retrospective studies [47]. Although there was a report from the Japanese multicenter randomized trial including 508 patients with resected malignancies of pancreas, bile duct, gallbladder, or ampulla of Vater between 1986 and 1992 [48], which showed OS improvement with mitomycin C plus 5-fluorouracil compared to observation in GBCA patients (n = 112), this study is limited by using old-fashioned chemotherapy regimen and lack of appropriate statistical consideration. A meta-analysis including 20 studies involving 6712 patients showed that there was a non-significant improvement in OS with any adjuvant treatment compared with surgery alone (pooled OR, 0.74; *p* = 0.06) [47]. Adjuvant treatment was shown to benefit patients with lymph-node–positive disease (OR, 0.49; *p* = 0.004) and R1 disease (OR, 0.36; *p* = 0.002). Although this analysis supported adjuvant treatment for resected BTC patients, it is difficult to use these findings to specifically guide adjuvant therapy for resected BTC patients because the patient population and specific adjuvant therapy strategies were diverse. Despite the lack of evidence derived from randomized trials, high recurrence rates after curative-intent resection have led to the wider use of adjuvant chemotherapy. The US National Cancer Database, a prospective, hospital-based cancer registry, showed that the proportion of patients receiving adjuvant chemotherapy has increased among patients with resected GBCA [49].

### 4.2. Randomized Trials

The baseline patient characteristics and key clinical outcomes are presented in Table 1 and Table 2, respectively.

#### 4.2.1. BILCAP Trial

The BILCAP trial was a randomized, open-label, phase 3 study conducted in 44 UK hospitals, which compared adjuvant capecitabine (1250 mg/m^2^ twice daily on days 1–14 of a 21-day cycle for eight cycles) with observation [13]. A total of 447 patients with CCA or muscle-invasive GBCA who had undergone macroscopically complete resection were enrolled between March 2006 and December 2014. At the time of the final analysis (March 6, 2017), 114 (51%) patients had died in the capecitabine group and 131 (58%) had died in the observation group. In the intention-to-treat (ITT) analysis, the median OS—the primary endpoint—was 51.1 months (95% confidence interval (CI), 34.6–59.1) in the capecitabine group and 36.4 months (95% CI, 29.7–44.5) in the observation group. The adjusted hazard ratio (HR) was 0.81 (95% CI, 0.63–1.04; *p* = 0.097). In the per-protocol (PP) analysis—including 430 patients—the median OS was 53 months (95% CI, 40-not reached) in the capecitabine group and 36 months (95% CI, 30–44) in the observation group with an adjusted HR of 0.75 (95% CI, 0.58–0.97; *p* = 0.028). In the ITT population, the median recurrence-free survival (RFS) was 24.4 months (95% CI, 18.6–35.9) in the capecitabine group and 17.5 months (95% CI, 12.0–23.8 months) in the observation group (adjusted HR, 0.75; 95% CI, 0.58–0.98; *p* = 0.033). In the PP population, the median RFS was 25.9 months (95% CI, 19.8–46.3 months) in the capecitabine group and 17.4 months (95% CI, 12.0–23.7) in the observation group (adjusted HR of 0.70 (95% CI, 0.54–0.92) and *p* = 0.0093). Although the primary endpoint of this study was not met in the ITT population, the prespecified PP analysis revealed significantly improved OS with capecitabine compared with observation. Based on the data from the BILCAP trial, capecitabine following curative-intent surgery is now regarded as a standard therapeutic strategy for resectable BTC patients.

#### 4.2.2. PRODIGE 12 Trial

The PRODIGE 12-ACCORD 18-UNICANCER GI trial was a randomized, open-label, phase 3 study comparing gemcitabine plus oxaliplatin (GEMOX: gemcitabine 1000 mg/m^2^ on day 1 and oxaliplatin 85 mg/m^2^ infused on day 2 of a 2-week cycle) for 12 cycles with observation for resected BTC [14]. A total of 196 patients who underwent curative-intent surgery were enrolled from 33 French hospitals between July 2009 and February 2014. The primary endpoints of this study were RFS and health-related quality of life (HRQOL). At the time of analysis, the events for RFS and deaths occurred in 126 and 82 patients, respectively. There was no significant between-group difference in RFS, as the median RFS was 30.4 months (95% CI, 15.4–43.0) in the GEMOX group and 18.5 months (95% CI, 12.6–38.2) in the observation group (HR, 0.88; 95% CI, 0.62–1.25; *p* = 0.48). The median time to definitive deterioration of global HRQOL did not differ between the two groups (31.8 months in the GEMOX group vs. 32.1 months in the observation group; [HR, 1.28; 95% CI, 0.73–2.26; *p* = 0.39]). The median OS was 75.8 months (95% CI, 34.4-incalculable) in the GEMOX group and 50.8 months (95% CI, 38.0-not estimable) in the observation group (HR, 1.08; 95% CI, 0.70–1.66; *p* = 0.74).

#### 4.2.3. BCAT Trial

The BCAT trial was a Japanese randomized open-label phase 3 trial investigating gemcitabine (1000 mg/m^2^ 3 weekly every 4 weeks for six cycles) as adjuvant therapy vs. observation for resected BTC patients [15]. This study only included EH-CCA (perihilar and distal CCA) patients. The primary endpoint was OS. A total of 226 patients were included from 48 Japanese hospitals between September 2007 and January 2011. There were no significant between-group differences in median OS (62.3 months in the gemcitabine group vs. 63.8 months in the observation group (HR, 1.01; 95% CI, 0.70–1.45; *p* = 0.964)) or median RFS (36.0 vs. 39.9 months (HR, 0.93; 95% CI, 0.66–1.32; *p* = 0.693)).

### 4.3. Interpretation of Conflicting Results and Future Perspectives

The conflicting results among these randomized studies reflect the heterogeneous disease characteristics of BTC. It is interesting that GEMOX, which is likely to have comparable efficacy to GemCis, is the current standard therapy for locally advanced and metastatic BTC patients based on ABC-02 trial. Compared with the BILCAP trial, the PRODIGE 12 trial seems to have been underpowered with a relatively small sample size (N = 194) [13,14]. There were also differences in baseline patient characteristics between the BILCAP and PRODIGE 12 trials, such as proportion of EH-CCA (35% vs. 28%), R1 resection status (38% vs. 13%), and lymph node positivity (47% vs. 38%), although it is not clear whether these differences affected the outcomes.

One of the potential explanations for the failure of previous randomized trials is the lumping together of all BTC tumor types. Even in the BILCAP trial, ITT analysis failed to yield statistically significant results. As surgical considerations and recurrence patterns vary by primary BTC tumor types, and as these might impact the clinical outcomes of adjuvant chemotherapy, investigations for each tumor type might be an effective strategy. Another potential explanation is the insufficient efficacy of the investigated adjuvant chemotherapy. Recent advances in the understanding of BTC have revealed that targeted therapy, such as FGFR or IDH-1, or immunotherapy, such as PD-1 or PD-L1 inhibitors, have activity against unresectable or metastatic BTC [12,50,51]. Future studies to incorporate these novel therapies in adjuvant treatment for resected BTC are needed.

### 4.4. Ongoing Adjuvant Chemotherapy Trials

There are ongoing large prospective randomized trials investigating adjuvant chemotherapy for resected BTC patients (Table 3). The ACTICCA-1 trial (NCT02170090) is a multinational trial comparing observation with GemCis for resected BTC. After the BILCAP trial findings were disseminated, the ACTICCA-1 study protocol was amended to include capecitabine in the control arm. An ongoing Chinese phase 3 trial (NCT02548195) is investigating GEMOX in comparison with capecitabine for resected IH-CCA patients. JCOG1202 (ASCOT) trial is a Japanese phase 3 trial comparing adjuvant S-1 with observation in resected biliary tract cancer. The STAMP trial (NCT03079427) is a Korean multi-center phase 2 trial investigating resected lymph-node–positive EH-CCA patients and comparing GemCis with capecitabine. This trial recently completed its target enrollment. Another Korean multi-center phase 3 trial (NCT04401709) comparing adjuvant capecitabine with gemcitabine–capecitabine combination therapy will be launched soon.

## 5. Neoadjuvant Chemotherapy

As a significant proportion of patients with BTC, particularly those with perihilar-CCA, are diagnosed with locally advanced disease, neoadjuvant chemotherapy may be an effective strategy for improving R0 resection rates and survival outcomes. However, there has been a lack of prospective clinical data on this subject. As intensive chemotherapy, such as with mFOLFIRINOX, has yielded promising data for borderline resectable or locally advanced pancreatic cancer [52,53], neoadjuvant chemotherapy may provide improved clinical outcomes for BTC patients. Triplet chemotherapy, such as GemCis plus nab-paclitaxel or S-1-irinotecan–oxaliplatin, have shown high response rates (40–50%) in single-arm studies, and these regimens may be as effective as neoadjuvant therapy [54,55]. A recent single-arm phase 2 study showed promising efficacy outcomes with durvalumab, anti-PD-L1 antibody, and GemCis for patients with unresectable or metastatic BTC, and this regimen is now under investigation (DEBATE: NCT04308174) as neoadjuvant therapy for resectable BTC (Table 3) [56].

## 6. Adjuvant Radiation Therapy

The role of adjuvant radiation therapy for BTC remains controversial due to a lack of data generated by large prospective randomized trials. However, as pathologically complete resection is often limited due to the locally invasive nature of disease and the proximity of tumors to adjacent critical structures, and because local recurrence is frequent even after curative-intent surgery, adjuvant radiation therapy has been used for resected BTC patients in daily practice [19].

In a previous large meta-analysis of adjuvant therapy [47], adjuvant chemotherapy and chemoradiotherapy were associated with greater benefits than adjuvant radiation therapy alone (odds ratio (OR) = 0.39 vs. 0.61 vs. 0.98, *p* = 0.02). The effect of adjuvant therapy was significant for patients with positive resection margin (OR = 0.36, *p* = 0.002), 63% of whom received radiation therapy alone. In another meta-analysis for EH-CCA and GBCA including 21 studies [57], the OS benefit of adjuvant radiation therapy (vs. no radiation therapy) was marginal for patients with negative resection margins (OR = 0.57, *p* = 0.08) and the adjuvant therapy group had a significantly higher 5-year OS rate among those with positive resection margins (OR = 0.40, *p* = 0.02) or positive lymph node metastases (OR = 0.15; *p* < 0.001). These findings were consistent with previous reports based on data from the US National Cancer Database and the Surveillance, Epidemiology, and End Results (SEER) database of the National Cancer Institute [58,59,60,61].

The Southwest Oncology Group (SWOG) S0809 trial was a multi-center prospective phase 2 trial to evaluate four cycles of gemcitabine followed by capecitabine-based chemoradiation therapy (54–59.4 Gy) for resected EH-CCA and GBCA (stage T2–4) with lymph node metastases or positive resection margins [62]. A total of 79 patients were enrolled, and 10 patients did not receive radiation therapy after chemotherapy. The median OS was 35 months, and the 2-year OS and disease-free survival (DFS) rates were 65% and 52%, respectively. The 2-year local recurrence rate was only 11%. The patients who did not receive radiation therapy and those who received radiation therapy with a deviation from protocol had higher local recurrence rates (30% and 42%, respectively). However, there were no significant differences in OS, DFS, and local recurrence at 2 years between the R0 and R1 resection groups, and these findings may suggest a beneficial effect of radiation therapy for R1 resection. The SWOG S0809 trial adopted a modern radiation therapy technique, including mostly intensity-modulated radiation therapy, and the severity and incidence of radiation therapy–related toxicity were acceptable. Amid a lack of high-level evidence supporting adjuvant radiation therapy for resected biliary tract cancer, this well-conducted prospective trial with a modern chemoradiation therapy protocol had a great influence on clinical practice and provided a well-established regimen for future trials investigating adjuvant radiation therapy.

Because of the lack of high-level evidence, current clinical practice guidelines recommended adjuvant radiation therapy for select patients with EH-CCA and GBCA after multidisciplinary discussion on the potential risks and benefits [63,64]. Recently published American Society of Clinical Oncology (ASCO) clinical practice guidelines suggest that adjuvant radiation therapy may be a reasonable option to reduce the risk of local recurrence and that patients with EH-CCA or GBCA with R1 resection may be offered chemoradiation therapy after a shared decision-making process [63]. Further efforts are required to identify the subgroup of patients who are most likely to benefit from adjuvant radiation therapy, and prospective randomized trials are needed.

## 7. Endpoints of Perioperative Therapy Clinical Trials

OS is a gold-standard hard efficacy endpoint of clinical trials investigating novel chemotherapeutic agents in advanced cancer patients. Therefore, OS is recommended to be the primary endpoint of perioperative therapy clinical trials in advanced BTC patients. BTC is relatively rare cancer type for which it is difficult to perform multiple large randomized trials; however, DFS may be considered as the surrogate endpoint to reduce the sample size, cost, and duration of clinical trials. Although a previous report has suggested that PFS may be the valid surrogate endpoint for OS in unresectable or metastatic BTC patients [65], there are no such data that show the correlation between DFS and OS in resected BTC. Further investigations are needed to define the optimal surrogate endpoints in the future clinical trials of perioperative therapy.

## 8. Conclusions

A multidisciplinary approach with shared decision-making is essential for the successful management of resectable BTC. With the development and standardization of surgical strategies for each BTC type (IH-CCA, perihilar CCA, distal CCA, and GBCA), future trials are needed to focus on specific biliary tract subsites. As novel chemotherapeutic agents and strategies, including targeted therapy and immunotherapy, have shown promising efficacy, there is a need for future research incorporating these novel therapies in the perioperative management of resectable BTC.

## Figures and Tables

**Table 1:** Baseline patient characteristics of prospective randomized adjuvant therapy trials.

Variables	BILCAP Trial		PRODIGE 12 Trial		BCAT Trial	
Treatment	CAP	Observation	GEMOX	Observation	GEM	Observation
Patient number	223	224	95	99	117	108
Age, median (range)	62 (55–68)	64 (55–69)	63 (33–83)	63 (41–80)	≥70 years: 56%	≥70 years: 43%
Gender, male/female	50%/50%	50%/50%	60%/40%	50%/50%	66%/34%	76%/24%
Primary tumor site						
IH-CCA	43 (19%)	41 (18%)	41 (43%)	45 (46%)	0	0
Perihilar CCA	65 (29%)	63 (28%)	10 (11%)	5 (5%)	51 (44%)	51 (47%)
Distal CCA	76 (34%)	80 (36%)	27 (28%)	28 (28%)	66 (56%)	57 (53%)
GBCA	39 (17%)	40 (18%)	17 (18%)	21 (21%)	0	0
Resection status						
R0	139 (62%)	140 (63%)	82 (86%)	87 (88%)	106 (91%)	94 (87%)
R1	84 (38%)	84 (38%)	13 (14%)	12 (12%)	11 (9%)	14 (13%)
Nodal status						
Negative	115 (52%)	121 (54%)	49 (52%)	48 (48%)	75 (64%)	72 (67%)
Positive	108 (48%)	102 (46%)	35 (37%)	36 (36%)	42 (36%)	36 (33%)
Missing	0	1 (<1%)	11 (12%)	15 (15%)		

CAP = capecitabine, CCA = cholangiocarcinoma, GBCA = gallbladder carcinoma, GEM = gemcitabine, GEMOX = gemcitabine plus oxaliplatin, IH-CCA = intrahepatic cholangiocarcinoma.

**Table 2:** Clinical outcomes of previous adjuvant chemotherapy trials.

Variables	BILCAP Trial (ITT)		BILCAP Trial (PP)		PRODIGE 12 Trial		BCAT Trial	
Treatment	CAP	Observation	CAP	Observation	GEMOX	Observation	GEM	Observation
Median OS, months (95% CI)	51.1 (34.6–59.1)	36.4 (29.7–44.5)	53 (40-not reached)	36 (30–44)	75.8 (34.4-not reached)	50.8 (38.0-not reached)	62.3	63.8
HR (95% CI)	0.81 (0.63–1.04)	*p* = 0.097	0.75 (0.58–0.97)	*p* = 0.028	1.08 (0.70–1.66)	*p* = 0.74	1.01 (0.70–1.45)	*p* = 0.964
Median RFS, months (95% CI)	24.4 (18.6–35.9)	17.5 (12.0–23.8)	25.9 (19.8–46.3)	17.4 (12.0–23.7)	30.4 (15.4–43.0)	18.5 (12.6–38.2)	36.0	39.9
HR (95% CI)	0.75 (0.58–0.98)	*p* = 0.033	0.70 (0.54–0.92)	*p* = 0.0093	0.88 (0.62–1.25)	0.48	0.93 (0.66–1.32)	*p* = 0.693

CAP = capecitabine, CI = confidence interval, GEM = gemcitabine, GEMOX = gemcitabine plus oxaliplatin, HR = hazard ratio, ITT = intention-to-treat, PP = per protocol, RFS = recurrence-free survival, OS = overall survival.

**Table 3:** Ongoing randomized trials for adjuvant and neoadjuvant chemotherapy.

Registered Number	Country	Phase	Number of Target Patients	Target Disease	Investigational Arm	Control Arm	Primary Endpoint
**Adjuvant chemotherapy**							
NCT02170090 (ACTICCA-1)	Germany/ Netherland/UK/ Austrailia	III	781	IH-CCA, EH-CCA, GBCA	GemCis	Observation → CAP	DFS
NCT02548195	China	III	286	IH-CCA	GEMOX	GEM	DFS
UMIN000011688 (JCOG1202, ASCOT)	Japan	III	440	IH-CCA, EH-CCA, GBCA, ampullary cancer	S-1	Observation	OS
NCT03079427 (STAMP)	Korea	II	100	LN-positive EH-CCA	GemCis	CAP	DFS
NCT04401709	Korea	III	490	IH-CCA, EH-CCA, GBCA	GEM+CAP	CAP	DFS
**Neoadjuvant chemotherapy**							
NCT03673072 (GAIN)	Germany	III	300	Incidental GBCA	GemCis	Upfront surgery	OS
NCT04308174 (DEBATE)	Korea	II	45	IH-CCA, EH-CCA, GBCA	Durvalumab + GemCis	GemCis	R0 resection rate

CAP = capecitabine, CCA = cholangiocarcinoma, DFS = disease-free survival, EH-CCA = extrahepatic cholangiocarcinoma, GBCA = gallbladder carcinoma, GEM = gemcitabine, GemCis = gemcitabine plus cisplatin, GEMOX = gemcitabine plus oxaliplatin, IH-CCA = intrahepatic cholangiocarcinoma, LN = lymph node, OS = overall survival.

## Data Availability

Not applicable.

## References

[1] Banales J.M., Marin J.J.G., Lamarca A., Rodrigues P.M., Khan S.A., Roberts L.R., Cardinale V., Carpino G., Andersen J.B., Braconi C. (2020). Cholangiocarcinoma 2020: The next horizon in mechanisms and management. Nat. Rev. Gastroenterol. Hepatol..

[2] Jung K.-W., Won Y.-J., Hong S., Kong H.-J., Lee E.S. (2020). Prediction of Cancer Incidence and Mortality in Korea, 2020. Cancer Res. Treat..

[3] Siegel R.L., Miller K.D., Jemal A. (2020). Cancer statistics, 2020. CA Cancer J. Clin..

[4] Valle J., Wasan H., Palmer D.H., Cunningham D., Anthoney A., Maraveyas A., Madhusudan S., Iveson T., Hughes S., Pereira S.P. (2010). Cisplatin plus Gemcitabine versus Gemcitabine for Biliary Tract Cancer. N. Engl. J. Med..

[5] Park J.O., Oh D.-Y., Hsu C., Chen J.-S., Chen L.-T., Orlando M., Kim J.S., Lim H.Y. (2015). Gemcitabine Plus Cisplatin for Advanced Biliary Tract Cancer: A Systematic Review. Cancer Res. Treat..

[6] Kim B.J., Hyung J., Yoo C., Park S.-J., Lee S.S., Song T.J., Seo D.W., Cho H., Ryoo B.-Y., Chang H.-M. (2017). Prognostic factors in patients with advanced biliary tract cancer treated with first-line gemcitabine plus cisplatin: Retrospective analysis of 740 patients. Cancer Chemother. Pharmacol..

[7] Okusaka T., Nakachi K., Fukutomi A., Mizuno N., Ohkawa S., Funakoshi A., Nagino M., Kondo S., Nagaoka S., Funai J. (2010). Gemcitabine alone or in combination with cisplatin in patients with biliary tract cancer: A comparative multicentre study in Japan. Br. J. Cancer.

[8] Kim B.J., Yoo C., Kim K.-P., Hyung J., Park S.J., Ryoo B.-Y., Chang H.-M. (2017). Efficacy of fluoropyrimidine-based chemotherapy in patients with advanced biliary tract cancer after failure of gemcitabine plus cisplatin: Retrospective analysis of 321 patients. Br. J. Cancer.

[9] Abou-Alfa G.K., Macarulla T., Javle M.M., Kelley R.K., Lubner S.J., Adeva J., Cleary J.M., Catenacci D.V., Borad M.J., Bridgewater J. (2020). Ivosidenib in IDH1-mutant, chemotherapy-refractory cholangiocarcinoma (ClarIDHy): A multicentre, randomised, double-blind, placebo-controlled, phase 3 study. Lancet Oncol..

[10] Abou-Alfa G.K., Sahai V., Hollebecque A., Vaccaro G., Melisi D., Al-Rajabi R., Paulson A.S., Borad M.J., Gallinson D., Murphy A.G. (2020). Pemigatinib for previously treated, locally advanced or metastatic cholangiocarcinoma: A multicentre, open-label, phase 2 study. Lancet Oncol..

[11] Jeong H., Jeong J.H., Kim K.-P., Lee S.S., Oh D.W., Park D.H., Song T.J., Park Y., Hong S.-M., Ryoo B.-Y. (2021). Feasibility of HER2-Targeted Therapy in Advanced Biliary Tract Cancer: A Prospective Pilot Study of Trastuzumab Biosimilar in Combination with Gemcitabine Plus Cisplatin. Cancers.

[12] Lamarca A., Barriuso J., McNamara M.G., Valle J.W. (2020). Molecular targeted therapies: Ready for “prime time” in biliary tract cancer. J. Hepatol..

[13] Primrose J.N., Fox R.P., Palmer D.H., Malik H.Z., Prasad R., Mirza D., Anthony A., Corrie P., Falk S., Finch-Jones M. (2019). Capecitabine compared with observation in resected biliary tract cancer (BILCAP): A randomised, controlled, multicentre, phase 3 study. Lancet Oncol..

[14] Edeline J., Benabdelghani M., Bertaut A., Watelet J., Hammel P., Joly J.-P., Boudjema K., Fartoux L., Bouhier-Leporrier K., Jouve J.-L. (2019). Gemcitabine and Oxaliplatin Chemotherapy or Surveillance in Resected Biliary Tract Cancer (PRODIGE 12-ACCORD 18-UNICANCER GI): A Randomized Phase III Study. J. Clin. Oncol..

[15] Ebata T., Hirano S., Konishi M., Uesaka K., Tsuchiya Y., Ohtsuka M., Kaneoka Y., Yamamoto M., Ambo Y., Shimizu Y. (2018). Randomized clinical trial of adjuvant gemcitabine chemotherapy versus observation in resected bile duct cancer. BJS.

[16] Si A., Li J., Yang Z., Xia Y., Yang T., Lei Z., Cheng Z., Pawlik T.M., Lau W.Y., Shen F. (2019). Impact of Anatomical Versus Non-anatomical Liver Resection on Short- and Long-Term Outcomes for Patients with Intrahepatic Cholangiocarcinoma. Ann. Surg. Oncol..

[17] Zhang X.-F., Bagante F., Chakedis J., Moris D., Beal E.W., Weiss M., Popescu I., Marques H.P., Aldrighetti L., Maithel S.K. (2017). Perioperative and Long-Term Outcome for Intrahepatic Cholangiocarcinoma: Impact of Major Versus Minor Hepatectomy. J. Gastrointest. Surg..

[18] De Jong M.C., Nathan H., Sotiropoulos G.C., Paul A., Alexandrescu S., Marques H., Pulitano C., Barroso E., Clary B.M., Aldrighetti L. (2011). Intrahepatic Cholangiocarcinoma: An International Multi-Institutional Analysis of Prognostic Factors and Lymph Node Assessment. J. Clin. Oncol..

[19] DeOliveira M.L., Cunningham S.C., Cameron J.L., Kamangar F., Winter J.M., Lillemoe K.D., Choti M.A., Yeo C.J., Schulick R.D. (2007). Cholangiocarcinoma. Ann. Surg..

[20] Bagante F., Gani F., Spolverato G., Xu L., Alexandrescu S., Marques H.P., Lamelas J., Aldrighetti L., Gamblin T.C., Maithel S.K. (2015). Intrahepatic Cholangiocarcinoma: Prognosis of Patients Who Did Not Undergo Lymphadenectomy. J. Am. Coll. Surg..

[21] Shimada M., Yamashita Y., Aishima S., Shirabe K., Takenaka K., Sugimachi K. (2001). Value of lymph node dissection during resection of intrahepatic cholangiocarcinoma. BJS.

[22] Zhang X., Chakedis J., Bagante F., Chen Q., Beal E.W., Lv Y., Weiss M., Popescu I., Marques H.P., Aldrighetti L. (2018). Trends in use of lymphadenectomy in surgery with curative intent for intrahepatic cholangiocarcinoma. BJS.

[23] Lee A.J., Chun Y.S. (2018). Intrahepatic cholangiocarcinoma: The AJCC/UICC 8th edition updates. Chin. Clin. Oncol..

[24] Okami J., Dono K., Sakon M., Tsujie M., Hayashi N., Fujiwara Y., Nagano H., Umeshita K., Nakamori S., Monden M. (2003). Patterns of regional lymph node involvement in intrahepatic cholangiocarcinoma of the left lobe. J. Gastrointest. Surg..

[25] Zhang X.-F., Xue F., Dong D.-H., Weiss M., Popescu I., Marques H.P., Aldrighetti L., Maithel S.K., Pulitano C., Bauer T.W. (2020). Number and Station of Lymph Node Metastasis After Curative-intent Resection of Intrahepatic Cholangiocarcinoma Impact Prognosis. Ann. Surg..

[26] FuksJean D., Regimbeau J.M., Le Treut Y.-P., Bachellier P., Raventos A., Pruvot F.-R., Chiche L., Farges O. (2011). Incidental Gallbladder Cancer by the AFC-GBC-2009 Study Group. World J. Surg..

[27] Lee S.E., Jang J.-Y., Kim S.-W., Han H.-S., Kim H.-J., Yun S.-S., Cho B.-H., Yu H.C., Lee W.J., Korean Pancreas Surgery Club (2014). Surgical Strategy for T1 Gallbladder Cancer: A Nationwide Multicenter Survey in South Korea. Ann. Surg. Oncol..

[28] Lee H., Choi D.W., Park J.Y., Youn S., Kwon W., Heo J.S., Choi S.H., Jang K.-T. (2014). Surgical Strategy for T2 Gallbladder Cancer According to Tumor Location. Ann. Surg. Oncol..

[29] Abramson M.A., Pandharipande P., Ruan D., Gold J.S., Whang E.E. (2009). Radical resection for T1b gallbladder cancer: A decision analysis. HPB.

[30] Kim H.S., Park J.W., Kim H., Han Y., Kwon W., Kim S.-W., Hwang Y.J., Kim S.G., Kwon H.J., Vinuela E. (2018). Optimal surgical treatment in patients with T1b gallbladder cancer: An international multicenter study. J. Hepato Biliary Pancreat. Sci..

[31] Kim N.H., Kim S.H., Choi G.H., Kang C.M., Kim K.S., Choi J.S., Lee W.J. (2013). Role of Cholecystectomy and Lymph Node Dissection in Patients with T2 Gallbladder Cancer. World J. Surg..

[32] D’Angelica M., Dalal K.M., DeMatteo R.P., Fong Y., Blumgart L.H., Jarnagin W.R. (2008). Analysis of the Extent of Resection for Adenocarcinoma of the Gallbladder. Ann. Surg. Oncol..

[33] Mizumoto R., Suzuki H. (1988). Surgical anatomy of the hepatic hilum with special reference to the caudate lobe. World J. Surg..

[34] Lee S.G., Song G.W., Hwang S., Ha T.Y., Moon D.B., Jung D.H., Kim K.H., Ahn C.S., Kim M.H., Sung K.B. (2010). Surgical treatment of hilar cholangiocarcinoma in the new era: The Asan experience. J. Hepato Biliary Pancreat. Sci..

[35] Shimizu H., Kimura F., Yoshidome H., Ohtsuka M., Kato A., Yoshitomi H., Furukawa K., Miyazaki M. (2010). Aggressive Surgical Resection for Hilar Cholangiocarcinoma of the Left-Side Predominance. Ann. Surg..

[36] Ito F., Cho C.S., Rikkers L.F., Weber S.M. (2009). Hilar Cholangiocarcinoma: Current Management. Ann. Surg..

[37] Ikeyama T., Nagino M., Oda K., Ebata T., Nishio H., Nimura Y. (2007). Surgical Approach to Bismuth Type I and II Hilar Cholangiocarcinomas. Ann. Surg..

[38] Nagino M., Kamiya J., Arai T., Nishio H., Ebata T., Nimura Y. (2006). “Anatomic” Right Hepatic Trisectionectomy (Extended Right Hepatectomy) With Caudate Lobectomy for Hilar Cholangiocarcinoma. Ann. Surg..

[39] Makuuchi M., Thai B.L., Takayasu K., Takayama T., Kosuge T., Gunvén P., Yamazaki S., Hasegawa H., Ozaki H. (1990). Preoperative portal embolization to increase safety of major hepatectomy for hilar bile duct carcinoma: A preliminary report. Surgery.

[40] Ribero D., Abdalla E.K., Madoff D.C., Donadon M., Loyer E.M., Vauthey J.-N. (2007). Portal vein embolization before major hepatectomy and its effects on regeneration, resectability and outcome. BJS.

[41] Farges O., Belghiti J., Kianmanesh R., Regimbeau J.M., Santoro R., Vilgrain V., Denys A., Sauvanet A. (2003). Portal Vein Embolization Before Right Hepatectomy. Ann. Surg..

[42] Hemming A.W., Reed A.I., Howard R.J., Fujita S., Hochwald S.N., Caridi J.G., Hawkins I.F., Vauthey J.-N. (2003). Preoperative Portal Vein Embolization for Extended Hepatectomy. Ann. Surg..

[43] Tran K.T.C., Smeenk H.G., van Eijck C.H.J., Kazemier G., Hop W.C., Greve J.W.G., Terpstra O.T., Zijlstra J.A., Klinkert P., Jeekel H. (2004). Pylorus Preserving Pancreaticoduodenectomy Versus Standard Whipple Procedure. Ann. Surg..

[44] Yeo C.J., Cameron J.L., Lillemoe K.D., Sohn T.A., Campbell K.A., Sauter P.K., Coleman J., Abrams R.A., Hruban R.H. (2002). Pancreaticoduodenectomy With or Without Distal Gastrectomy and Extended Retroperitoneal Lymphadenectomy for Periampullary Adenocarcinoma, Part 2. Ann. Surg..

[45] Sasaki R., Takahashi M., Funato O., Nitta H., Murakami M., Kawamura H., Suto T., Kanno S., Saito K. (2001). Prognostic significance of lymph node involvement in middle and distal bile duct cancer. Surgery.

[46] Jarnagin W.R., Ruo L., Little S.A., Klimstra D.S., I Dangelica M., DeMatteo R.P., Wagman R., Blumgart L.H., Fong Y. (2003). Patterns of initial disease recurrence after resection of gallbladder carcinoma and hilar cholangiocarcinoma. Cancer.

[47] Horgan A.M., Amir E., Walter T., Knox J.J. (2012). Adjuvant Therapy in the Treatment of Biliary Tract Cancer: A Systematic Review and Meta-Analysis. J. Clin. Oncol..

[48] Takada T., Amano H., Yasuda H., Nimura Y., Matsushiro T., Kato H., Nagakawa T., Nakayama T., Study Group of Surgical Adjuvant Therapy for Carcinomas of the Pancreas and Biliary Tract (2002). Is postoperative adjuvant chemotherapy useful for gallbladder carcinoma?. Cancer.

[49] Cao H.S.T., Zhang Q., Sada Y.H., Chai C., Curley S.A., Massarweh N.N., Curley S.A. (2018). The role of surgery and adjuvant therapy in lymph node-positive cancers of the gallbladder and intrahepatic bile ducts. Cancer.

[50] Chae H., Kim D., Yoo C., Kim K.-P., Jeong J.H., Chang H.-M., Lee S.S., Park D.H., Song T.J., Hwang S. (2019). Therapeutic relevance of targeted sequencing in management of patients with advanced biliary tract cancer: DNA damage repair gene mutations as a predictive biomarker. Eur. J. Cancer.

[51] Yoo C., Oh D.-Y., Choi H.J., Kudo M., Ueno M., Kondo S., Chen L.-T., Osada M., Helwig C., Dussault I. (2019). Phase I study of bintrafusp alfa, a bifunctional fusion protein targeting TGF-β and PD-L1, in patients with pretreated biliary tract cancer. J. Immunother. Cancer.

[52] Yoo C., Hwang I., Song T.J., Lee S.S., Jeong J.H., Park D.H., Seo D.W., Lee S.K., Kim M.-H., Byun J.H. (2020). FOLFIRINOX in borderline resectable and locally advanced unresectable pancreatic adenocarcinoma. Ther. Adv. Med Oncol..

[53] Yoo C., Lee S.S., Song K.B., Jeong J.H., Hyung J., Park D.H., Song T.J., Seo D.W., Lee S.K., Kim M.-H. (2020). Neoadjuvant modified FOLFIRINOX followed by postoperative gemcitabine in borderline resectable pancreatic adenocarcinoma: A Phase 2 study for clinical and biomarker analysis. Br. J. Cancer.

[54] Shroff R.T., Javle M.M., Xiao L., Kaseb A.O., Varadhachary G.R., Wolff R.A., Raghav K.P.S., Iwasaki M., Masci P., Ramanathan R.K. (2019). Gemcitabine, Cisplatin, and nab-Paclitaxel for the Treatment of Advanced Biliary Tract Cancers. JAMA Oncol..

[55] Yoo C., Han B., Kim H.S., Kim K.-P., Kim D., Jeong J.H., Lee J.-L., Kim T.W., Kim J.H., Choi D.R. (2018). Multicenter Phase II Study of Oxaliplatin, Irinotecan, and S-1 as First-line Treatment for Patients with Recurrent or Metastatic Biliary Tract Cancer. Cancer Res. Treat..

[56] Oh D.-Y., Lee K.-H., Lee D.-W., Kim T.Y., Bang J.-H., Nam A.-R., Lee Y., Zhang Q., Rebelatto M., Li W. (2020). Phase II study assessing tolerability, efficacy, and biomarkers for durvalumab (D) ± tremelimumab (T) and gemcitabine/cisplatin (GemCis) in chemo-naïve advanced biliary tract cancer (aBTC). J. Clin. Oncol..

[57] Ren B., Guo Q., Yang Y., Liu L., Wei S., Chen W., Tian Y. (2020). A meta-analysis of the efficacy of postoperative adjuvant radiotherapy versus no radiotherapy for extrahepatic cholangiocarcinoma and gallbladder carcinoma. Radiat. Oncol..

[58] Wang S.J., Fuller C.D., Kim J.-S., Sittig D.F., Jr C.R.T., Ravdin P.M. (2008). Prediction Model for Estimating the Survival Benefit of Adjuvant Radiotherapy for Gallbladder Cancer. J. Clin. Oncol..

[59] Mitin T., Enestvedt C.K., Jemal A., Sineshaw H.M. (2017). Limited Use of Adjuvant Therapy in Patients With Resected Gallbladder Cancer Despite a Strong Association With Survival. J. Natl. Cancer Inst..

[60] Hoehn R.S., Wima K., Ertel A.E., Meier A., Ahmad S.A., Shah S.A., Abbott D.E. (2015). Adjuvant Chemotherapy and Radiation Therapy is Associated with Improved Survival for Patients with Extrahepatic Cholangiocarcinoma. Ann. Surg. Oncol..

[61] Nassour I., Mokdad A.A., Porembka M.R., Choti M.A., Polanco P.M., Mansour J.C., Minter R.M., Wang S.C., Yopp A.C. (2018). Adjuvant Therapy Is Associated with Improved Survival in Resected Perihilar Cholangiocarcinoma: A Propensity Matched Study. Ann. Surg. Oncol..

[62] Ben-Josef E., Guthrie K.A., El-Khoueiry A.B., Corless C.L., Zalupski M.M., Lowy A.M., Jr C.R.T., Alberts S.R., Dawson L.A., Micetich K.C. (2015). SWOG S0809: A Phase II Intergroup Trial of Adjuvant Capecitabine and Gemcitabine Followed by Radiotherapy and Concurrent Capecitabine in Extrahepatic Cholangiocarcinoma and Gallbladder Carcinoma. J. Clin. Oncol..

[63] Shroff R.T., Kennedy E.B., Bachini M., Bekaii-Saab T., Crane C., Edeline J., El-Khoueiry A., Feng M., Katz M.H., Primrose J. (2019). Adjuvant Therapy for Resected Biliary Tract Cancer: ASCO Clinical Practice Guideline. J. Clin. Oncol..

[64] Valle J.W., Borbath I., Khan S.A., Huguet F., Gruenberger T., Arnold D. (2016). Biliary cancer: ESMO Clinical Practice Guidelines for diagnosis, treatment and follow-up. Ann. Oncol..

[65] Moriwaki T., Yamamoto Y., Gosho M., Kobayashi M., Sugaya A., Yamada T., Endo S., Hyodo I. (2016). Correlations of survival with progression-free survival, response rate, and disease control rate in advanced biliary tract cancer: A meta-analysis of randomised trials of first-line chemotherapy. Br. J. Cancer.

